# Influence of growth rate on the physiological response of marine *Synechococcus* to phosphate limitation

**DOI:** 10.3389/fmicb.2015.00085

**Published:** 2015-02-11

**Authors:** Cécilia B. Kretz, Doug W. Bell, Debra A. Lomas, Michael W. Lomas, Adam C. Martiny

**Affiliations:** ^1^Department of Ecology and Evolutionary Biology, University of California IrvineIrvine, CA, USA; ^2^Marine Science Program, School of Earth, Ocean and Environment, University of South CarolinaColumbia, SC, USA; ^3^Bigelow Laboratory for Ocean SciencesEast Boothbay, ME, USA; ^4^Department of Earth System Science, University of California IrvineIrvine, CA, USA

**Keywords:** marine cyanobacteria, chemostat, redfield ratio, elemental stoichiometry

## Abstract

Phosphate (P) is an important nutrient potentially limiting for primary productivity, yet, we currently know little about the relationship between growth rate and physiological response to P limitation in abundant marine Cyanobacteria. Thus, the aim of this research was to determine how variation in growth rate affected the physiology of marine *Synechococcus* WH8102 and CC9311 when growing under high N:P conditions. Experiments were carried out in chemostats with a media input N:P of 441 and we estimated the half saturation concentration for growth under P limiting conditions (*K*_*s,p*_) and cellular C:N:P ratios. The *K*_*s,p*_ values were the lowest measured for any phytoplankton and on par with ambient P concentrations in oligotrophic regions. We also observed that both strains were able draw down P below 3 nM. Both *K*_*s,p*_ and drawdown concentration were lower for the open ocean vs. coastal *Synechococcus* strain, which may be linked to differences in P acquisition genes in these strains. Cellular C:P and N:P ratios were significantly higher in relation to the Redfield ratio for both *Synechococcus* strains but we saw no difference in these ratios among growth rates or strains. These results demonstrate that *Synechococcus* can proliferate under very low P conditions and also that genetically different strains have unique physiological responses to P limitation.

## Introduction

Marine microorganisms are a major component of global nutrient cycles and contribute roughly one-half of global net primary production (Field, 1998). Phosphorus plays a key role in regulating ocean primary productivity (Tyrell, 1999) and can limit phytoplankton growth in some regions. Phosphorus can be found in oligotrophic waters in a variety of forms including particulate inorganic phosphorus (Pi) and dissolved organic phosphorus (DOP). In the Sargasso Sea, most of the total dissolved phosphorus is found as DOP (Ammerman et al., 2003) and Pi concentrations in surface waters can be lower than 1 nM (Wu, 2000; Lomas et al., 2010). However, there are regional differences in the concentrations of DOP and SRP and with various sources available, different strategies for P assimilation and allocation can be employed by picophytoplankton (Moore et al., 2005; Martiny et al., 2006; Van Mooy and Devol, 2008; Casey et al., 2009).

Rates of growth and nutrient uptake, as a function of nutrient concentration are commonly described by hyperbolic equations. These include the Monod equation relating growth and nutrient availability parameterized by *K*_*s*_ and μ_*max*_, and the Michaelis–Menten equation relating uptake and nutrient concentration parameterized by *K*_*m*_ and *V*_*max*_ (Michaelis and Menten, 1913; Monod, 1949). Furthermore, the Monod equation is sometimes modified to include a parameter *c*, which represents the minimal concentration of substrate to sustain cell growth (Nyholm, 1977; DiToro, 1980; Button, 1985). We currently have limited quantitative knowledge of these parameters for many abundant ocean phytoplankton lineages and there are considerable variations between the few estimates available. *Synechococcus K*_*s,p*_ or *K*_*m,p*_values have been reported to range from 14 to 67,000 nM (Donald et al., 1997; Ikeya et al., 1997; Timmermans et al., 2005). Similarly, *K*_*s,p*_ or *K*_*m,p*_ for *Prochlorococcus* ranges from 8 to 18 nM at Station ALOHA (Bjorkman et al., 2012) to 900 nM in culture (Krumhardt et al., 2013) which infers a low phosphate affinity for *Prochlorococcus*. Some of these *K*-values are high considering that both *Prochlorococcus* and *Synechococcus* can grow at phosphorus concentrations below 0.5 nM in the Sargasso Sea (Lomas et al., 2010). Thus, we hypothesized that specific lineages may be better adapted for growth under low P conditions and characterized by *K*_*s*_ values closer to ambient P concentrations.

Another key feature related to nutrient stress in phytoplankton is the cellular C:N:P stoichiometry, which can vary depending on growth and nutrient availability. The growth rate hypothesis (GRH) states that cells become enriched in P (and thus, have lower cellular N:P) when growing at high rates (Elser et al., 1996, 2003). Another set of models suggests that the elemental ratio is constant at low growth rates but approaches an optimal value when cells are growing near μ_*max*_ (Klausmeier et al., 2004; Bonachela et al., 2013). Moreover, current biogeochemical ocean models assume a fixed molar C:N:P (Redfield's ratio) (Moore et al., 2004; Aumont and Bopp, 2006; Follows et al., 2007) however, variability in elemental ratios and growth rates has been reported in the field (Liu et al., 1998; Doney et al., 2009; Martiny et al., 2013). Also, the plasticity of phytoplankton stoichiometry in relation to growth has been investigated in a few phytoplankton species (Finkel et al., 2006; Quigg et al., 2011) but little is known about such plasticity in *Synechococcus. Synechococcus* is one of the major contributors to biomass of plankton communities in oligotrophic gyres (Lomas et al., 2010) and thus, knowing the stoichiometric ratios for specific species and how it relates to growth rate will help us better understand patterns of elemental ratios in the ocean and parametize biogeochemical ocean models.

Here, we use *Synechococcus* cultures to determine the half-saturation concentration for growth under P limiting conditions (*K*_*s,p*_), the minimal drawdown concentration for phosphate *c*, as well as the elemental ratios for two *Synechococcus* strains. One was isolated from an open ocean environment (WH8102) and the other one is a coastal strain (CC9311) isolated from coastal ocean environment (Palenik et al., 2003, 2006). We hypothesize that at low P concentrations, *Synechococcus* WH8102 will have a higher affinity for phosphate as a substrate than CC9311, and thus a lower *K*_*s,p*_, and that the cellular C:P and N:P of *Synechococcus* will be greater than the Redfield ratio (Redfield, 1934) but decline with growth rate (Bertilsson et al., 2003; Sterner et al., 2008; Martiny et al., 2013).

## Materials and method

### Culturing conditions

*Synechococcus* WH8102 and CC9311 were grown in continuous cultures in a chemostat at 24°C (±1), 35 μE/m^2^/s light intensity at 12 h light-dark cycles. All cultures were grown in a modified SN media (Waterbury et al., 1986) which was composed of 75% 0.22 μm filtered autoclaved seawater and 25% milli-Q water. We amended the media with the following nutrients: 2 μM final concentration NaH_2_P0_4_.H_2_O, 882 μM final concentration NaNO_3_, 15 μM final concentration Na_2_EDTA.2H_2_O, 95 μM final concentration Na_2_CO_3_ and lastly 1 ml of a trace metal solution per liter of media. The trace metal solution was composed of 6.25 g of citric Acid.H_2_O, 6 g of ferric ammonium citrate, 1.4 g MnCl_2_.4H_2_O, 0.39 g Na_2_MoO_4_.2H_2_0, 0.025 g Co(NO_3_)_2_.6H_2_0 and 0.222 g ZnSO_4_.7H_2_O. per liter. All media solutions were vacuumed filtered on a 0.22 μm filter since some of the components could not be autoclaved and stored in acid washed glassware at 4°C.

### Chemostat system

Two replicated (4 L) vessels containing each strain were maintained by adding a constant supply of 2 μM inorganic phosphate and 882 μM of nitrate (Figure S1). The environmental variables: temperature and light levels were held constant throughout the experiments and growth rate was manipulated by changing the speed of the pump every 2 weeks, which in turn changed the dilution rate. We changed the dilution rate 3 times and the resulting growth rate averaged 0.7 day^−1^, 0.3 day^−1^, and 0.2 day^−1^ per strain.

### Estimation of the half saturation growth rate concentration (*K*_*s,p*_)

From the measured dilution, associated growth rate and phosphate concentration (SRP), we estimated the associated growth rate in the vessels. Then from the growth rate and the measured phosphate concentration (Table S1), we estimated *K*_*s,p*_ for each vessel and strain by fitting both a Monod function (using Sigmaplot v.12, Systat Software, San Jose, CA) and a custom function with a critical threshold and a fixed μ_*max*_. The equation used was as follows: *y* = [μ_*max*_ (*x* – *c*) / (*K*_*s*_ + (*x* – *c*)], where μ_*max*_ was fixed at 0.75 day^−1^ (Moore et al., 1995) and *c* represents the phosphate drawdown value or the minimum P concentration needed for cell growth (DiToro, 1980).

### Soluble reactive phosphate and particulate organic phosphate

Fifty ml of cells were collected 3 times a week from each outlet tube and soluble reactive phosphate (SRP) of the media was measured using the method developed by Murphy and Riley and adapted from Karl et al. and Lomas et al. (Murphy and Riley, 1962; Karl and Tien, 1992; Lomas et al., 2010). We measured the phosphate concentration of the vessel to determine which concentration the cells experience. We also collected 50 ml for particulate organic phosphate (cell quota for P), which were subsequently filtered onto a pre-combusted (5 h at 500°C) HCl and milli-Q water rinsed 25 mm GF/F filter (Whatman, Florham Park, NJ) filter using acid washed glassware and stored at −20°C until analysis. Then 2 ml of 0.017 M MgSO4 was added to each filter, and they were placed in acid washed scintillation vials and dried at 60°C overnight. After drying, the filters were baked at 500°C for 2 h. After cooling, 5 ml of 0.2 M HCl was added to each scintillation vial, and they were heated for 30 min at 80–90°C. Once at room temperature, the supernatant was decanted into a 15 ml acid washed glass centrifuge tube and 1 ml of the ammonium molybdate mixed reagent was added (Solorzano and Sharp, 1980; Lomas et al., 2010). The tube was inverted to mix and centrifuged at 4500 rpm for 1 min. Optical density was read at 885 nm using Genesys 10vis spectrophotometer (Thermo Scientific, Waltham, MA) and phosphate concentrations were calculated from an asymptotic regression of absorbance vs. known concentrations of potassium phosphate standards and corrected for media blanks.

### Particulate organic carbon and nitrogen measurements

Fifteen ml of cells were collected from each chemostat vessel and filtered onto pre-combusted HCl and milli-Q water rinsed 13 mm GF/F filters using acid-washed glassware. The filters were allowed to dry at 60°C for 1 day and stored at −20°C until further analysis. Each filter was carefully packed into a tin cup. Carbon and nitrogen percentages on the filters were determined using a CHN analyser (Thermo Finnigan EA 1112, Bremen, Germany). The percent of C/N was calculated from chromatogram area using atropine standards and corrected for media blanks.

### Statistical analysis

We used Wilcoxon signed rank tests to test whether our measured C:N:P ratios differed from the Redfield ratio and Mann-Whitney rank sum test to test the differences between strains. Spearman's rank correlations were used to assess if growth rate and stoichiometry were correlated.

## Results

We determined the half-saturation concentration for growth, *K*_*s,p*_ value and the elemental ratios for two strains of *Synechococcus* (WH8102 and CC9311) in a P-limited chemostat setup (Figure S1) at three different growth rates in order to determine how variation in growth rate affected their physiology.

### Determination of *K*_*s,p*_

**Figure 1:**
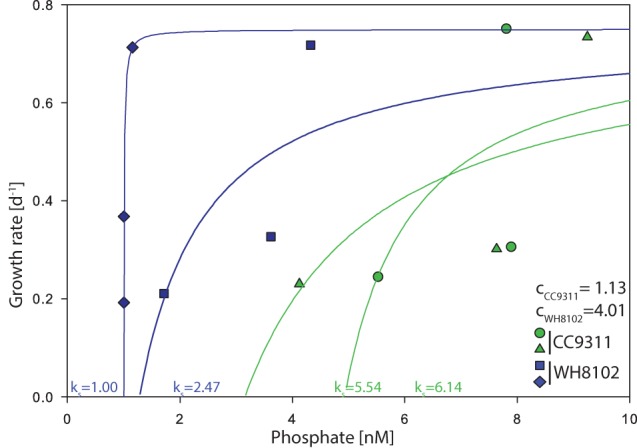
**Growth rate as a function of external phosphate concentration for *Synechococcus* strains WH8102 and CC9311 in 4 chemostat vessels**. The half saturation concentration for growth (K_*s,p*_) values were determined using a modified Monod's growth rate kinetics equation. The blue curves represent the replicated WH8102 strains while the green curves represent the replicated CC9311 curves. The draw down concentrations *c* are noted for WH8102 and for CC9311.

Every 2 weeks, we manipulated the dilution rate and growth rate was then estimated for each chemostat vessel (Table 1). Growth rate averaged to 0.2 day^−1^, 0.3 day^−1^, and 0.7 day^−1^ per strain for the 3 different intervals. The inorganic phosphate concentration (SRP) in each vessel ranged between 1.0 and 9.2 nM for the duration of the experiment. Using these measurements and assuming a maximal growth rate of 0.75 day^−1^, we estimated the half saturation concentration for growth under P limiting conditions *K*_*s,p*_. *K*_*s,p*_ ranged from 5.5 to 6.1 nM for CC9311 and 0.8 to 2.5 nM for WH8102 (Figure 1 and Table 1). Thus, WH8102 appeared to have a lower *K*_*s,p*_ in comparison to CC9311. From the modified Monod equation we also estimated the parameter *c*, which represents the minimal amount of substrate to sustain cell growth. *c*-Values ranged from 0.9 to 1.2nM (averaged 1.1 nM) for WH8102 and ranged from 3.1 to 4.9 nM (averaged 4.0 nM) for CC9311. Therefore, WH8102 was also capable of drawing down the residual P concentration to a lower value compared to CC9311. The fit of the resulting Monod curves ranged between *R*^2^ = 0.568 to *R*^2^ = 0.889 for WH8102 and *R*^2^ = 0.338 to *R*^2^ = 505 for CC9311.

**Table 1:** **Summary of measured and calculated parameters, including averaged growth rate (at the 3 time intervals, T1, T2, and T3), K_s_ and K′_s_ (ranges for the K_s,p_ value), *c*, the drawdown value for phosphate, averaged soluble reactive phosphorus (SRP) per time interval, and molar averaged C:N:P ratios and the average molar ratios per strains using the raw measurements**.

**Strain**	**Replicate**	**Growth rate (day^−1^)**			**K_*s*_**	**K′_*s*_**	**c**	**Molar ratio**			**Average molar ratio per strains**			**SRP (nM)**		
		**T1**	**T2**	**T3**				**N:P**	**C:P**	**C:N**	**N:P**	**C:P**	**C:N**	**T1**	**T2**	**T3**
WH8102	1	0.2	0.3	0.7	2.5	2.4	1.2	31.3	186.8	6.1	26.2	152	6.7	1.7	3.6	4.3
WH8102	2	0.2	0.4	0.7	1	0.8	0.9	16.2	92.9	6.7				1	1	1.2
CC9311	1	0.2	0.3	0.7	6.1	5.1	3.1	29	144.1	5.1	31.5	174	4.9	4.1	7.6	9.2
CC9311	2	0.2	0.3	0.8	5.5	5.3	4.9	33.4	166.8	5				5.5	7.9	7.8

T1, T2, and T3 are respectively: 0.7 day^−1^, 0.3 day^−1^, and 0.2 day^−^.

### Cellular elemental stoichiometry

**Figure 2:**
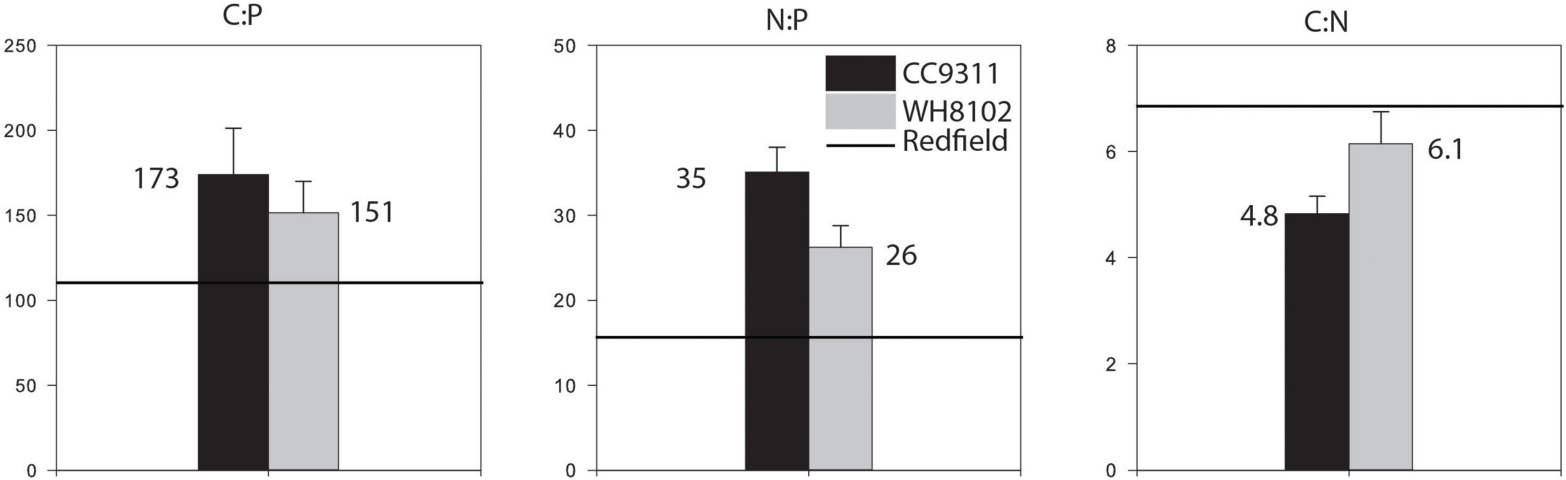
**Comparison of cellular C:N:P ratios for strains WH8102 and CC9311 and the Redfield ratio (106:16:1)**. The average between the strains are represented. The error bar represent the standard error between the technical replicates. C:N:P between strains were not statistically different (Mann–Whitney rank sum test, *P* > 0.05). C:P and N:P ratios were greater than Redfield (Wilcoxon signed rank test, *P* < 0.001). C:N for *Synechococcus* CC9311 was lower than Redfield (Wilcoxon signed rank test, *P* < 0.001) but not statistically different for WH8102 (*P* > 0.05).

**Figure 3:**
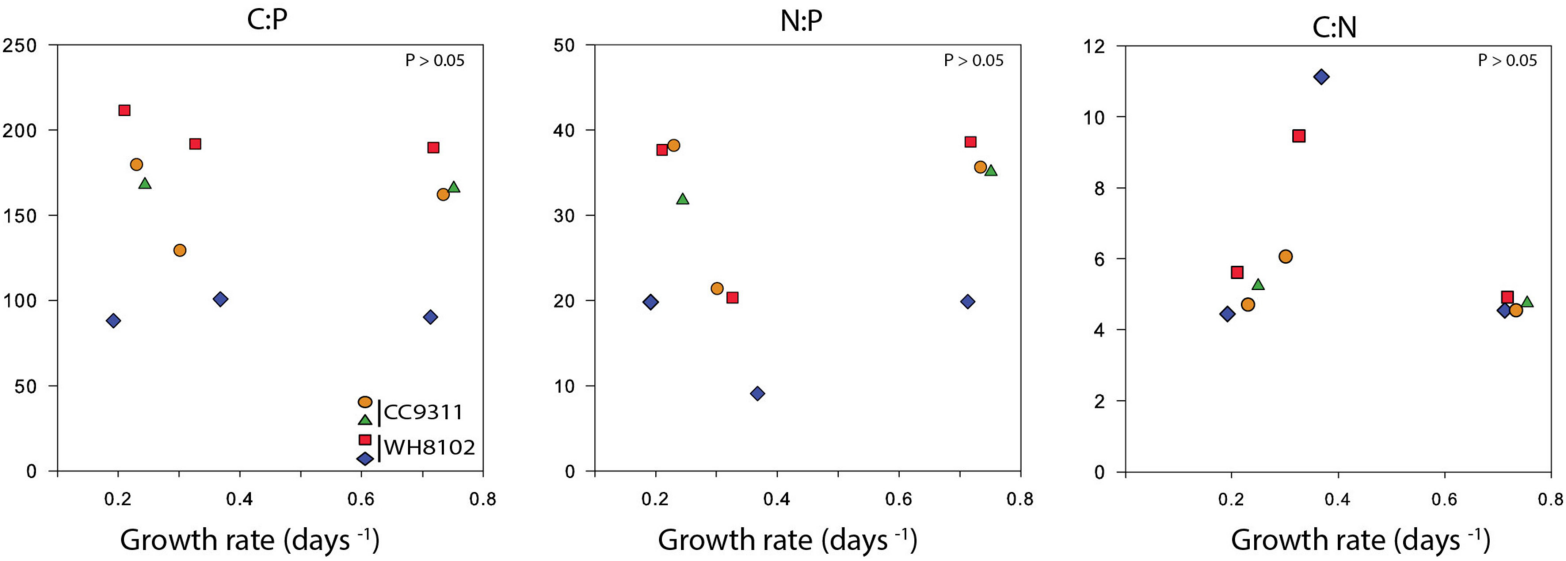
**Cellular C:N:P ratios as a function of growth rate for each *Synechococcus* strain in the chemostat**. The correlations between growth rate and the different ratios were determined using Spearman's rank correlation coefficient (*P* > 0.05).

We next estimated the cellular nutrient ratios. The average C:P ratio was 151.5 and 173.9 and the average N:P ratio was 26.2 and 35.1 for WH8102 and CC9311 respectively, (for timepoints and vessel averages see Table 1 and Table S2). However, we only had 2 dilution time points for one of the replicates of CC9311 due to experimental complications. Nevertheless, the C:P and N:P ratios were significantly higher than the Redfield ratio for both strains (molar C:N:P = 106:16:1) (*P*_wilcoxon_ < 0.05), whereas the C:N ratios were lower (although only significantly for CC9311) (C:N ratios of 4.83 and 6.15 respectively, (*P*_wilcoxon_ < 0.05). However, the ratios for CC9311 and WH8102 were not significantly different (Figure 2) (*P*_Mann−Whitney_ > 0.05). We also examined the relationship between growth rate and cellular ratios but observed no correlation (*P*_Spearman_ > 0.05 for all strains and ratios) (Figure 3). Moreover, elemental ratios did not change systematically over time, which excludes an effect of the experimental design on the values measured for cellular C:N:P (Figure S2).

## Discussion

Using chemostats and high sensitivity nutrient measurements, we observe that both *Synechococcus* strains are able to grow at near maximum rates at P concentrations below 10 nM. This includes a *K*_*s,p*_ value below 10 nM as well as the ability to draw down P to less than 3nM. The *K*_*s,p*_ values are substantially lower than previously observed (Donald et al., 1997; Ikeya et al., 1997; Timmermans et al., 2005) but close to the ambient P concentration in the Western North Atlantic Ocean (Lomas et al., 2010). We further observe that the open ocean strain WH8102 had a lower *K*_*s,p*_ and *c*. This is consistent with the idea and possible cause that the open ocean strain is able to proliferate under very low P conditions by transcribing genes for high affinity uptake transporters, alkaline phosphatases regulon *pho*A, and P uptake proteins (Moore et al., 2005; Tai and Palenik, 2009). In comparison, *Synechococcus* CC9311 lacks a phosphate sensor-response regulator system and an alkaline phosphatase as well as having fewer genes for periplasmic phosphate binding proteins used in the transport system (Palenik et al., 2006). These predicted differences in gene content for phosphate acquisition and regulation have been linked to the availability of phosphate in the ocean environment (Palenik et al., 2006). Although we do not determine a mechanism between genome content and growth physiology under low P concentrations, our observations are consistent with the hypothesis that WH8102 is better adapted to growth under low P conditions. The fit of the Monod curves can be explained by the number of time points that we had for the experiment. However, each of those time points varied between 2 and 3 weeks.

Based on previous studies (Bertilsson et al., 2003; Martiny et al., 2013), it has been reported that molar stoichiometric ratios for *Synechococcus* can be greater than Redfield proportions. In support, we measured C:P and N:P ratios of 151–173 and 26–35, respectively. Moreover, we do not identify a link between growth rate and C:N:P which deviates from the growth rate hypothesis (GRH) model (Figure 3) but since our results indicate that the elemental ratios are constant at low growth rate, the data may support Klausmeier's model (Klausmeier et al., 2004; Bonachela et al., 2013). This is based on the idea that at maximum growth rate, there is only one possible C:N:P however, when cells are not required to grow optimally, the ratios can vary according to the supply of N:P. Also, the *Synechococcus* strains we used appeared capable of maxing out the cellular ratios at the analyzed growth rate, which would, in return, cancel out any effect of growth rate. Moreover, recent studies have estimated the contribution of RNA to total cellular phosphate pool and found that RNA was only a small fraction (Zimmerman et al., 2014a,b). Thus, a change in growth and rRNA does not appear to have an important impact on cellular N:P. The range of C:N:P measurements and some disparity between our replicated vessels are due to variations in the flow of the chemostat. This system can easily develop clogs in the tubing, which must be removed to sustain the flow of media. Fluctuations in flow rate affect the nutrient concentrations at the time of measurement, leading to a greater variance in observations. We also attribute the poor fit of some of the Monod curves to variations in the chemostat.

When comparing the C:N:P ratios between strains of *Synechococcus*, we find no significant differences between WH8102 and CC9311, even though previous studies have suggested that taxonomic composition will influence elemental stoichiometry (Twining et al., 2004; Arrigo, 2005; Price, 2005) however, these studies did not compare strains within genera. Few studies have compared the elemental ratios of strains of *Synechococcus* and *Prochlorococcus* and found that they were similar within strains (Bertilsson et al., 2003); however, another study using different strains of *Synechococcus* found a marked difference in C:N:P between two strains suggesting some strain-specific differences (Heldal et al., 2003). A recent study has investigated variations in C:N:P in heterotrophic bacteria and suggested that strain-level diversity was an important driver (Zimmerman et al., 2014a). However, for our study, we compared within the same genus, it is therefore possible that the taxonomic composition needed to be further expanded to include comparisons between different species rather than only different strains.

Although our results need to be expanded with the addition of more strains of *Synechococcus*, they suggest that genomic variation could lead to functional differences in *Synechococcus* populations. These functional differences may include variations in the ability to assimilate P under low nutrient conditions. The obtained measurements for C:N:P and *K*_*s,p*_ values could be of use for modelers in order to improve the parameterization of growth under nutrient limited conditions in ocean biogeochemical models since most current models assume a constant C:N:P (Redfield ratio) and it is not an accurate representation of certain regions of the ocean. It would also enable us to more accurately predict future changes in ocean nutrient cycles.

### Conflict of interest statement

The authors declare that the research was conducted in the absence of any commercial or financial relationships that could be construed as a potential conflict of interest.
