# Supplementary material for: Influence of growth rate on the physiological response of marine Synechococcus to phosphate limitation
Source: Front Microbiol. 2015 Feb 11;6:85. doi: 10.3389/fmicb.2015.00085 (PMC4324148; doi:10.3389/fmicb.2015.00085)
Supplement: Supplementary file 1 [file Table1.PDF]

Table S1: Dissolved inorganic phosphate (SRP) throughout the experiment in the replicated vessels for CC9311 and WH8102.

Strain	Vessel	Date	P (nM)	Strain	Vessel	Date	P (nM)
CC9311	1	22-Feb-10	11.25	WH8102	1	22-Feb-10	0.83
CC9311	1	24-Feb-10	2.43	WH8102	1	24-Feb-10	14.46
CC9311	1	26-Feb-10	13.65	WH8102	1	26-Feb-10	1.00
CC9311	1	1-Mar-10	9.65	WH8102	1	1-Mar-10	1.00
CC9311	1	3-Mar-10	1.00	WH8102	1	3-Mar-10	1.00
CC9311	1	5-Mar-10	11.25	WH8102	1	5-Mar-10	0.83
CC9311	1	8-Mar-10	2.43	WH8102	1	8-Mar-10	1.00
CC9311	1	10-Mar-10	1.00	WH8102	1	10-Mar-10	4.03
CC9311	1	12-Mar-10	8.04	WH8102	1	12-Mar-10	16.86
CC9311	1	15-Mar-10	1.00	WH8102	1	19-Mar-10	1.00
CC9311	1	17-Mar-10	19.27	WH8102	1	22-Mar-10	1.00
CC9311	1	19-Mar-10	15.26	WH8102	1	24-Mar-10	16.06
CC9311	1	22-Mar-10	1.63	WH8102	1	26-Mar-10	8.84
CC9311	1	24-Mar-10	1.00	WH8102	2	22-Feb-10	1.00
CC9311	1	26-Mar-10	1.00	WH8102	2	24-Feb-10	1.63
CC9311	2	19-Feb-10	5.64	WH8102	2	26-Feb-10	1.00
CC9311	2	22-Feb-10	5.64	WH8102	2	1-Mar-10	1.00
CC9311	2	24-Feb-10	3.23	WH8102	2	3-Mar-10	4.03
CC9311	2	26-Feb-10	6.44	WH8102	2	5-Mar-10	1.00
CC9311	2	1-Mar-10	1.00	WH8102	2	8-Mar-10	1.00
CC9311	2	3-Mar-10	1.00	WH8102	2	10-Mar-10	1.00
CC9311	2	5-Mar-10	1.00	WH8102	2	15-Mar-10	1.00
CC9311	2	8-Mar-10	0.03	WH8102	2	17-Mar-10	1.00
CC9311	2	15-Mar-10	20.07	WH8102	2	19-Mar-10	1.00
CC9311	2	19-Mar-10	21.67	WH8102	2	22-Mar-10	1.00
CC9311	2	22-Mar-10	1.00	WH8102	2	24-Mar-10	1.00
CC9311	2	24-Mar-10	1.00	WH8102	2	26-Mar-10	1.00
