# Supplementary material for: Influence of growth rate on the physiological response of marine Synechococcus to phosphate limitation
Source: Front Microbiol. 2015 Feb 11;6:85. doi: 10.3389/fmicb.2015.00085 (PMC4324148; doi:10.3389/fmicb.2015.00085)
Supplement: Supplementary file 2 [file Table2.PDF]

Table S2:Average C:N:P values per dilution time points per replicated vessels
t1, t2 and t3 correspond to the growth rate time periods used during the experiment
t1=0.7 day^{-1}, t2=0.3 day^{-1}, t3= 0.2 day^{-1}

Strain	Replicate	N:P			C:P			C:N		
		t1	t2	t3	t1	t2	t3	t1	t2	t3
**WH8102**	1	34.4	38.9	20.3	166.1	228.1	181.4	4.6	5.7	9.1
**WH8102**	2	23.0	20.3	11.9	110.1	89.0	119.0	4.6	4.5	11.8
**CC9311**	1	30.5	43.6	20.5	140.9	227.0	120.6	4.6	4.5	6.0
**CC9311**	2	44.6	32.3		206.7	160.6		4.5	5.1	
