# Supplementary figures and images for: Influence of growth rate on the physiological response of marine Synechococcus to phosphate limitation
Source: Front Microbiol. 2015 Feb 11;6:85. doi: 10.3389/fmicb.2015.00085 (PMC4324148; doi:10.3389/fmicb.2015.00085)

Figure S1: Diagram representing the chemostat experiment

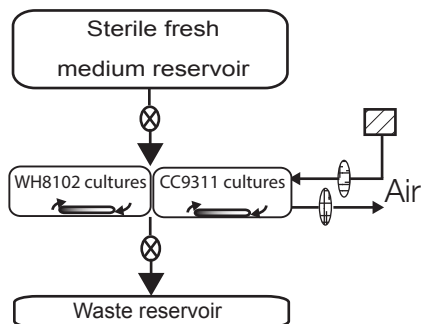

Supplement: Supplementary file 3 [file Image1.PDF]
