# Supplementary material for: Influence of growth rate on the physiological response of marine Synechococcus to phosphate limitation
Source: Front Microbiol. 2015 Feb 11;6:85. doi: 10.3389/fmicb.2015.00085 (PMC4324148; doi:10.3389/fmicb.2015.00085)

Figure S2: C:N:P as a function of time during the experiment. Growth rates have been added for each time period.

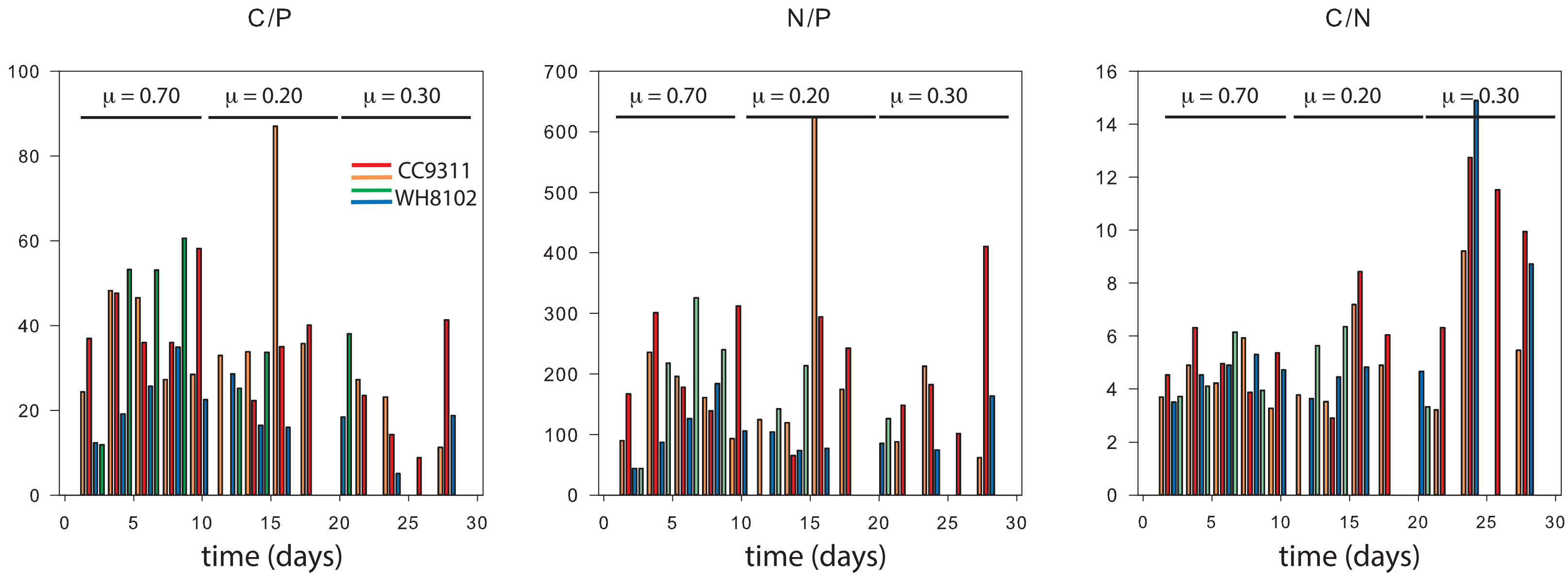

Supplement: Supplementary file 4 [file Image2.PDF]
